# Quantification of tumour heterogeneity and glucose metabolism on pre-chemoradiation PET/CT predicts survival in anal cancer

**DOI:** 10.1186/1470-7330-15-S1-S11

**Published:** 2015-10-02

**Authors:** A Afaq, B Ganeshan, T Grenader, G Azzopardi, R Endozo, J Bridgewater, A Groves

**Affiliations:** 1 University College London, Gower Street, London, WC1E 6BT, UK

## Aim

To investigate CT Texture Analysis (CTTA, marker of tumour heterogeneity) and metabolic information from PET as potential prognostic biomarkers in patients with anal squamous cell carcinoma (SCC) treated with chemoradiotherapy (CRT).

## Methods

42 patients (median age 60.2 years, range 36.8-80.2; 19 males) with anal SCC, who received CRT with pre-treatment 18F-FDG PET/CT were retrospectively reviewed. CTTA was performed on the largest tumour diameter CT image using TexRAD software. CTTA used a filtration-histogram technique, extracting fine, medium and coarse texture features, followed by quantification of histogram parameters. SUVmax, metabolic tumour volume(MTV) and total lesion glycolysis(TLG) were measured using Metavol software. Mean follow-up period was 39.6 months. Kaplan-Meier analysis assessed the relationships between CTTA, PET and clinical parameters against progression free survival (PFS) and overall survival (OS).

## Results

Mean PFS was 65.6 months (95% CI:55.0-76.1) and mean OS was 73.0 (95% CI:63.9-82.2) months.

Higher pre-treatment CTTA kurtosis was consistently a significant predictor of PFS and OS respectively (best at medium-scale: p=0.007, p=0.022).

PET and clinical parameters significantly predicted PFS and OS respectively included SUVmax (p=0.0138, p=0.0038), MTV(p=0.0049, p=0.005), TLG (p=0.0027, p=0.0011), tumour stage (p=0.01, p=0.0003) and nodal stage (p=0.002, p=0.0003).

## Conclusion

This study identified kurtosis, as a marker of tumour heterogeneity on pre-treatment CTTA was associated with poorer OS and PFS. SUVmax, MTV and TLG were also all predictors of poorer survival as were higher stage tumours. A multi-parametric approach with these features may provide prognostic information in patients with anal SCC undergoing treatment with chemoradiotherapy.

